# Taxonomic changes in some predominantly Palaearctic distributed genera of Drymini (Heteroptera, Rhyparochromidae)

**DOI:** 10.3897/zookeys.319.4465

**Published:** 2013-07-30

**Authors:** Előd Kondorosy

**Affiliations:** 1 Pannon University, Department of Animal Science, H-8360 Deák F. u. 16., Keszthely, Hungary

**Keywords:** Rhyparochromidae, Drymini, new synonyms, new genus, Palaearctic Region, Oriental Region, Australia

## Abstract

The history of the taxonomic research of Rhyparochromidae and especially Drymini is briefly reviewed. Two new species level synonyms are proposed: *Taphropeltus javanus* Bergroth, 1916, **syn. n.** = *Taphropeltus australis* Bergroth, 1916, **syn. n.** = *Brentiscerus putoni* (Buchanan White, 1878). A monotypic new genus, *Malipatilius* **gen. n.** (type species: *Scolopostethus forticornis* Gross, 1965 from Australia) is established.

## Introduction

**Figure 1.**
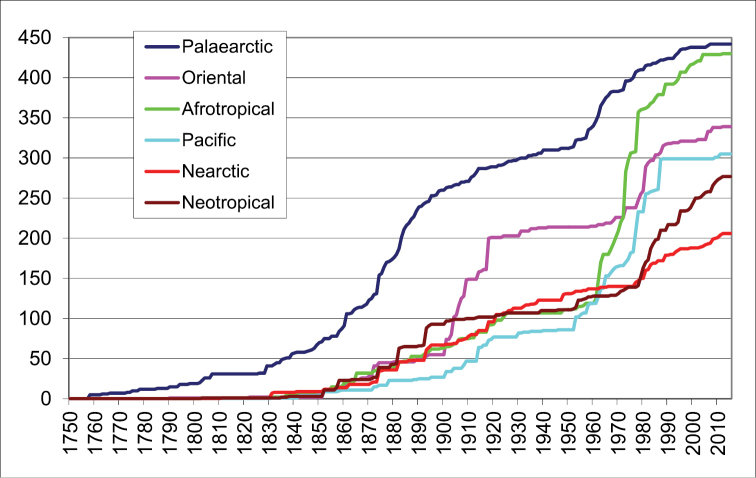
Number of described valid species of the family Rhyparochromidae from the main zoogeographical regions.

The knowledge on the taxonomy of the true bugs (Hemiptera: Heteroptera) and among them the family Rhyparochromidae developed unevenly during the past more than 250 years (Fig. 1). From Linnaeus till the end of the 19th century the European fauna was most intensively studied. The fauna of the temperate and tropical Americas started to receive more attention from the second half of the century; among others, the work of C. Stål, W. L. Distant and P. R. Uhler is outstanding. The first twenty years of the 20th century was the Golden Age of the research on the Oriental fauna thanks primarily to E. Bergroth, G. Breddin and W. L. Distant). Between the first and second World Wars the research intensity decreased globally except of the Nearctic region where important works were published by H. G. Barber and others. In the 1960’s–1980’s the research underwent an active period, and a high number of new taxa was described especially from the Afrotropical and Australian (+ Pacific) Regions, but also from other regions; the activity of J. A. Slater and G. G. E. Scudder, furthermore A. C. Eyles, R. E. Linnavuori, M. Malipatil and T. E. Woodward was especially significant. In the last twenty years the descriptive activity slackened again.

The species occurring in more than one zoogeographical regions are included only once in Fig. 1: in the region of the type locality. Therefore the known species number in each region is more or less higher than the listed one (Palaearctic: 475:442, Oriental: 456:339, Afrotropical: 544:430, Australian: 360:305, Nearctic: 258:206, Neotropical: 393:277). These differences refer to the many common species between certain regions (especially Palaearctic and Oriental or Afrotropical; and Nearctic and Neotropical regions).

The situation in respect of the tribe Drymini is similar to the general trends of Rhyparochromidae. This tribe is of worldwide distribution but in the Western Hemisphere the species richness is much lower (and only one species reaches the Neotropical area in Middle America). A good characterization of the world distribution of the Drymini (and of the other Rhyparochromidae) was given by Slater (1986) in his excellent work but this is the only tribe which dispersion is not evaluated in Slater’s interpretations.

Members of Drymini are usually moderately vagile. This might partly explain the fact that only a few species are distributed in more than one zoogeographical area. Most of these species occur in China where they center the Oriental areas but more or less broadly extend to the neighbouring Palaearctic territories or vice versa. Therefore the species numbers described from the each region are only slightly lower than the actual number of the species known in the respective areas (Palaearctic: 83:87, Oriental: 71:79, Afrotropical: 69:72, Australian: 25:26, Nearctic: 34:36, Neotropical: 1:1).

**Figure 2.**
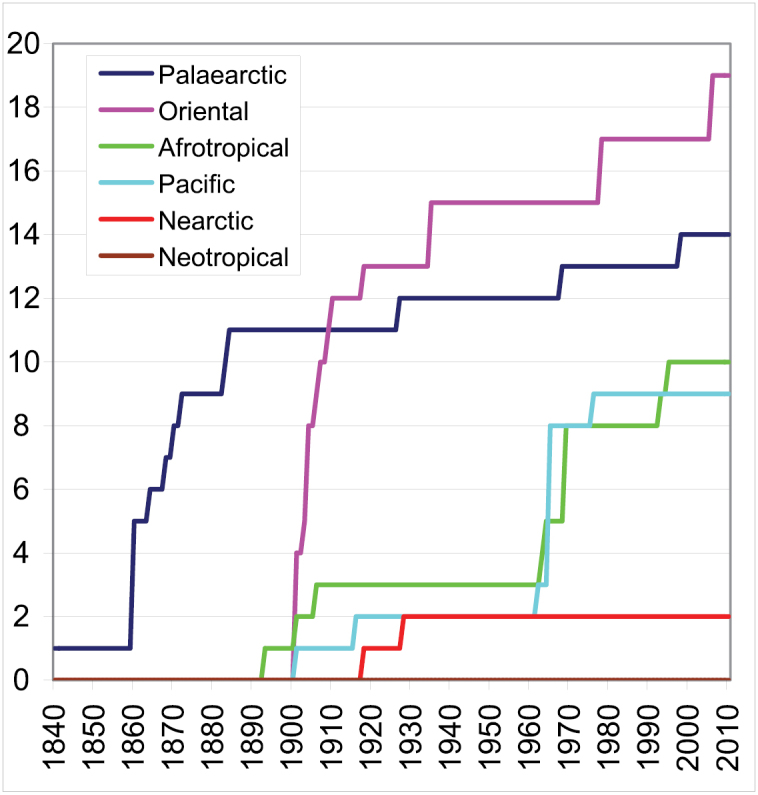
Number of described valid genera of the tribe Drymini from the main zoogeographical regions.

Examining the history of the genus and species description in Drymini, the following things can be observed (Fig. 2): during the 19^th^ century 12 genera of Drymini were described, 11 of them from the Palaearctic Region. Of the 65 discovered species 51 have Palaearctic distribution. In the first quarter of the 20^th^ century 18 genera and 42 species were described (among them 14 genera and 30 species from southeast Asia). Before World War II the Nearctic species were most intensively studied. The knowledge of the Drymini of the Australian Region was developed extremely thanks to Gross (1965): he described 7 of the known 9 genera and 20 of the 25 species. The Afrotropical region was most intensively studied betwen 1950 and 2000; 7 of the 10 known genera and 65 of the 69 known species were described during these fifty years.

The species described before the activity of F. X. Fieber were placed in large “general” genera as *Lygaeus* Fabricius, 1794, *Pachymerus* Lepeletier & Serville, 1825, or *Rhyparochromus* Hahn, 1826. Fieber proposed six genera in the Palaearctic Drymini. For the coming few decades the European genera were used to accommodate several new extrapalaearctic species; since most species occurring in the Nearctic Region belong to shared genera it was justified in many cases. Bergroth and Distant were the first to describe several extrapalaearctic genera in the beginning of the 20^th^ century. As a result of their activity, the use of the Palaearctic genera became more restricted. Currently several of these have already been transferred to other genera, but some species have remained “forgotten” or are of uncertain status.

The aim of this paper is to correct some of these incorrect combinations.

## Material and methods

Type and non-type specimens of Drymini of the following institutions were examined: Natural History Museum, LondonBMNH; Finnish Museum of Natural History, HelsinkiFMNH; Hungarian Natural History Museum, Budapest, HungaryHNHM; Moravian Museum, Brno, Czech RepublicMMBC; National Museum of Natural History (Naturalis), Leiden, the NederlandsRMNH; Natural History Museum, ViennaNHMW; Tirolese Regional Museum (Ferdinandeum), InnsbruckTLMF; Zoological Museum, AmsterdamZMAN, and Natural History Museum, BerlinMFNB.

## Results

### Distribution of Palaearctic genera of Drymini

As it was pointed out before, most species of Drymini are restricted to a single zoogeographical region. Similarly, most of the genera are also restricted to a single region. As an example, *Ischnocoris* Fieber, 1860, *Notochilus* Fieber, 1864, *Orsillodes* Puton, 1884, and *Thaumastopus* Fieber, 1870 are of exclusively Palaearctic distribution.

*Hidakacoris* Tomokuni, 1998 is currently known only from Japan, but there are some undescribed Oriental species which belong to this genus too.

All more widely distributed Palaearctic genera (13) extend to the Oriental region; 7 of them are found only in these two regions. Palaearctic genera containing species extending to Oriental areas are *Gastrodes* Westwood, 1840, *Lamproplax* Douglas & Scott, 1868, and *Trichodrymus* Lindberg, 1927. It is also frequent that an Oriental or pantropical genus has some species inhabiting marginal areas of the Palaearctic Region, most frequently China and Japan. Such genera are *Appolonius* Distant, 1901, *Mizaldus* Distant, 1901 (*Neomizaldus* Scudder, 1968, is probably a junior synonym of the latter), *Paradieuches* Distant, 1883, *Potamiaena* Distant, 1910, and *Retoka* China, 1935.

*Drymus* Fieber, 1860, is a genus centered in the Holarctic but also having some described and a few undescribed Oriental species.

### *Taphropeltus* Stål, 1872

*Taphropeltus* is a predominantly Palaearctic genus which currently contains one exotic species, too (two other Palaearctic species are reaching the northern part of the Afrotropical region). The Australian *Taphropeltus australis* Bergroth, 1916, was originally included in this genus (Bergroth 1916b). Gross (1965) described *Isopeltus* Gross, 1965, and designated *Taphropeltus australis* as its type species. Subsequently Slater (1976) synonymized *Isopeltus* with *Brentiscerus* Scudder, 1962 (type species: *Scolopostethus putoni* Buchanan White, 1878).

The othertropical *Taphropeltus* species is *Taphropeltus javanus* Bergroth, 1916, described from Java, Indonesia (Bergroth 1916a).

Both *Taphropeltus australis* and *Taphropeltus javanus* show similarity to the Palaearctic members of *Taphropeltus*, but they are readily distinguished from the true *Taphropeltus* species among others by having a more strongly developed pronotal collar and three rows of claval punctures. Based on the original descriptions only, Scudder (1962) presumed that the two species are congeneric. Because no important differences between the Australian and Javanese specimens could be found which would justify considering them as representing two different species, furthermore there specimens were seen from islands between the two type localities (Bali, Flores, Sumba, New Guinea: none of them was previously known as inhabited by any *Taphropeltus* species), the two species are considered as conspecific.

Both species were described in 1916. Both of the two journal issues contain explicit information about the date of the publication: the description of *Taphropeltus javanus* is dated to 12 September 1916, while the article describing *Taphropeltus australis* was published during October of the same year.

Furthermore, I compared the lectotype and additional non-type specimens of *Brentiscerus putoni* (Buchanan White, 1878), which is described from New Zealand, with the mentioned species. They are virtually identical. As a conclusion, the following nomenclatural changes are required:

#### Brentiscerus putoni

(Buchanan White, 1878)

http://species-id.net/wiki/Brentiscerus_putoni

Scolopostethus putoni Buchanan White, 1878: 75. Syntypes (♂, ♀): New Zealand; BMNH!
Taphropeltus javanus Bergroth, 1906a [12 Sep.]: 220. Syntype (s): [Indonesia:] Java, Mt. Tengger; lost? **syn. n.**
Taphropeltus australis Bergroth, 1906b [Oct.]: 13. Syntype (s): Australia: Victoria; lost? **syn. n.**

##### Type material examined.

***Scolopostethus putoni*. Lectotype** (designated by Scudder 1967): round label with purple margin LECTOTYPE // round label with red margin TYPE // hw: New Zealand // Scolopostethus / putoni B.W. // Brentiscerus / putoni (Wk.) / ExDr. 77 // printed: Pres. by / Perth Museum / B. M. 1953-629. //pink hw. Scolopostethus / putoni White 1878 / G.G.E. Scudder 1965 / LECTOTPYE (female, BMNH). **Paralectotypes**: round label with blue margin PARALECTOTYPE // round label with yellow margin COTYPE // hw: New Zealand // Scolopostethus / putoni B.W. // printed: Pres. by / Perth Museum / B. M. 1953-629. (1 male, 2 females all with the same labels, BMNH).

The types of ***Taphropeltus australis*** and ***Taphropeltus javanus*** are probably lost, no references mentioning them could be traced and they could not be found in FMNH where most of Bergroth’s collection is deposited. Taxonomic decisions were made based by examination of non-type specimens from Australia, New Guinea and Indonesia, respectively.

##### Additional material examined.

**INDONESIA.** Dammerman / O. Soemba / 700 m 249 / Kananggar / v. 1925 (1 male, RMNH); Dammerman / Idjen 1850 m / Ongop-ongop / 19. V. 1924 / No. 17 (RMNH); Banjoewangi / JAVA 1909 / MacGillavry (1 female, HNHM); INDONESIA: centr. Java / Pokalongan Reg., Bandar / 1050 m / 2.1998., leg. S. Jakl (1 female, NHMW); IDN-Bali Isl. / Bedugul reg. 1300m / Tamblingan lak.N.R. / S. Jakl lg., 3.2005 (1 female, MMBC); Sunda Exp. Rensch / W.-Flores / Rana Mêsé / 20.–30.6.1927 (1 male, MFNB); Sumba (E) / Luku-Melolo N. R. / 550 m, VII. 2005 / leg. S. Jakl (2 ex., NHMW). **PAPUA NEW GUINEA.** New Guinea / Mt. Kaindi / 2400 m / 15-16. IV. 1965 // Nr. 34 / Coll. Balogh et / Szent-Ivány (1 female, HNHM); Austr. New Guinea / Wau 1250 m / 10.-20. XI. 1972 / J. v. d. Vecht (1 male, ZMAN); Museum Leiden / Neth. New Guinea Exp. / Star Range 1260 m / Sibil / 15. VI. 1959 // Taphropeltus 3 (handwriting) (1 female, RMNH); **AUSTRALIA**. N.S.W. / Cassilis “Kuloo” / Station 710 m / 31°50'9"S, 150°8'E // 25.X.2000 / Hung. Entom. Exped. / leg. A. Podlussány, G. Hangay & I. Rozner (1 male, HNHM); N.S.W. / Karai State Forest / Kookaburra, 943 m / 31°1'4"S, 152°20'2"E // 27–28.X.2000 / Hung. Entom. Exped. / leg. A. Podlussány, G. Hangay & I. Rozner (1 female, HNHM); N.S.W., Putty / Road, Cases Courvert / 10–11.I.2006 leg. G. Hangay, I. Rozner & A. Podlussány (1 male, 2 female, HNHM); N.S.W. / Milton, 21.I.2006 / leg. A. Podlussány, G. Hangay & I. Rozner (1 female, HNHM); New South Wales / J.P. Duffels // Eucalyptus / forest // 48 km N of Singleton / 15 I 1983 (1 female, ZMAN). **NEW ZEALAND**. C. Darwin / 85–119. (1 male, BMNH); (handwriting): Kaitaia NZ / 1 VIII 23 / JG Myers // Base of prairie grass // (printed): J. G. Myers Coll. B.M. 1937-789. (1 male, BMNH).

The population of *Brentiscerus putoni* in New Zealand possibly originates from Australia, where all congeners are native. There are no autochthonous Drymini species in New Zealand, only some introduced species occur, as *Brentiscerus putoni*, *Grossander major* (Gross, 1965) and *Paradrymus exilirostris* Bergroth, 1916 (Malipatil 1977). Since it feeds on *Eucalyptus* seeds (Gross 1965), *Brentiscerus putoni* likely was introduced with *Eucalyptus* trees.

The other species of the genus *Taphropeltus* species which are partly of extrapalaearctic distribution are *Taphropeltus nervosus* (Fieber, 1861) and *Taphropeltus ornatus* Linnavuori, 1978. Both of these species are morphologically rather distinct from the type species, *Taphropeltus hamulatus* Thomson, 1870, and the other known Palaearctic members of the genus. It is sure that at least *Taphropeltus ornatus* belongs to another genus, as it also was suggested by Péricart (1999). This problem needs further investigation.

### *Eremocoris* Fieber, 1860, and *Scolopostethus* Fieber, 1860

Although the West Palaearctic species of this complex are easy to classify into one of the two genera, *Eremocoris* and *Scolopostethus* are morphologically very close to each other. Some of the described species and also certain undescribed species from the Afrotropical and Oriental Regions are morphologically transitional between *Eremocoris* and *Scolopostethus*. E.g., the African *Scolopostethus maumus* Scudder, 1962, is apparently very closely related to *Eremocoris africanus* Slater, 1964. The possible synonymy of them was already suggested by Slater (1972).

Species currently placed to *Scolopostethus* live in all major zoogeographic regions, with many undescribed Oriental species. The Australian *Scolopostethus forticornis* Gross, 1965, belongs to a different, so far undescribed genus which is described below as new. Each of the African *Scolopostethus daulias* Linnavuori, 1978 and *Scolopostethus kilimandjariensis* Scudder, 1962 represent another undescribed genus. *Scolopostethus daulias* seems to be related with *Taphropeltus ornatus* Linnavuori, 1978, but their relationship needs further investigation. *Scolopostethus kilimandjariensis* belongs to a new genus but its description must be done in frames of a comprehensive study on all other Afrotropical members of the *Scolopostethus*–*Eremocoris* complex.

#### Malipatilius gen. n.

urn:lsid:zoobank.org:act:1B42F8DE-6D15-4B14-BE9B-BD02DEAF79FC

http://species-id.net/wiki/Malipatilius

##### Type species.

*Scolopostethus forticornis* Gross, 1965, by present designation.

##### Description.

**Figure 3.**
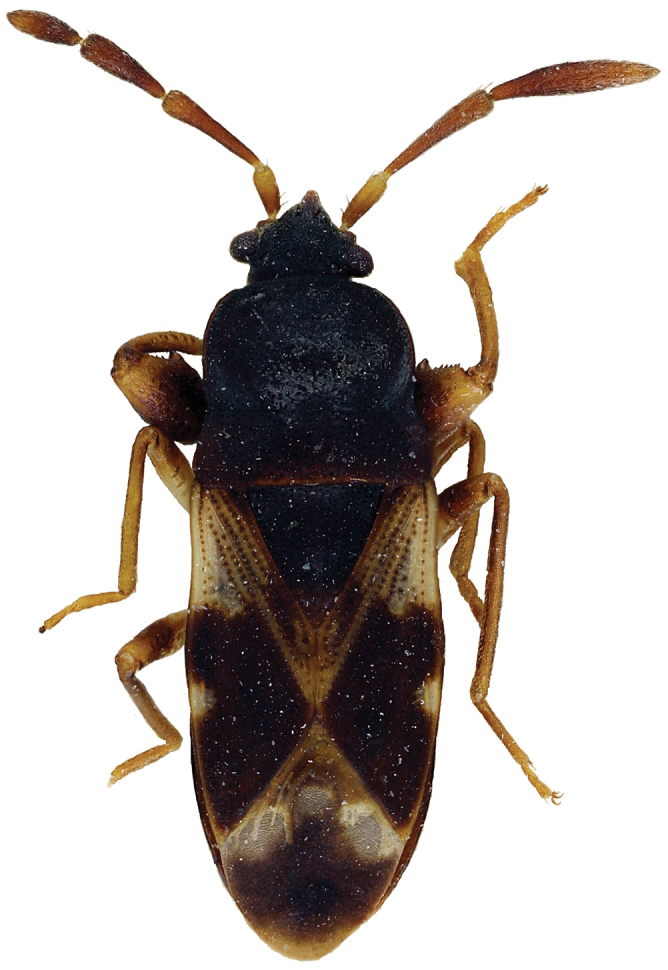
*Malipatilius forticornis* (Gross, 1965), new combination.

*Body* elongate oval, dull, extensively punctate, dorsally glabrous (Fig. 3).

*Head* pentagonal, with dense fine punctures. Eyes small, very prominent. Ocelli well developed, located very far from each other, near the eyes. Antenniferous tubercle curved laterally. *Antenna* very robust, subclavate.

*Pronotum* without anterior collar, transversal furrow deep, disk densely punctured. Anterior and posterior margins straight, lateral margin concave, explanate but not widened at transversal furrow. Anterior lobe more globose in male, lateral margin partially parallel here. *Scutellum* elevated at middle. *Fore wing*. Clavus with 3 regular rows of punctures. Corium evenly and densely punctate, nearly parallel, costal margin only slightly concave subbasally, apical margin straight. *Thoracic sternum* punctate except submedian parts of mesosternum. *Legs* robust, fore femur strongly incrassate, especially in male, with two rows of spines and a very large spine in inner row.

*Abdomen* with dense decumbent pilosity, lateral portion of intersegmental suture between sternites IV–V curved anteriorly, not reaching lateral margin and sublateral furrow; trichobothrial pattern as typical in Drymini.

##### Included species.

The genus is apparently not monotypic, because besides of the type species very probably congeneric specimens were seen at least from Java, Kalimantan and the New Hebrides.

##### Discussion:

**Figure 4.**
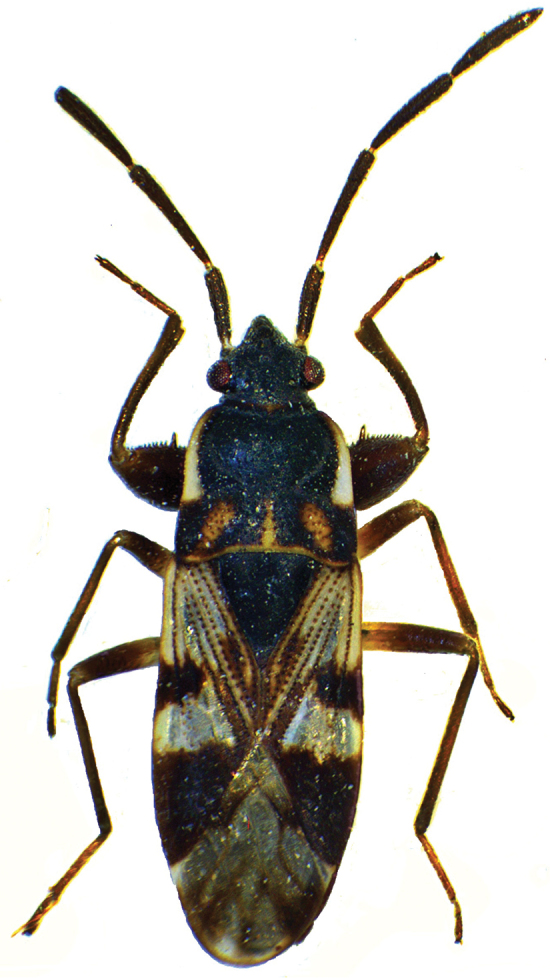
*Scolopostethus ornandus* Distant, 1904.

The type species of *Malipatilius* gen. n. was originally placed into the genus *Scolopostethus*. The diagnostic characters of the two genera are presented in Table 1. A typical *Scolopostethus* species, *Scolopostethus ornandus* Distant, 1904 is imaged for comparison on Fig. 4. *Faelicianus* Bergroth, 1918, is perhaps the sister genus of *Malipatilius* gen. n. This genus has a pale wide lateral carina on pronotum, which is broadened at transversal impression, therefore the pronotum is evenly convex laterally. The antenna is also slender, much more than even in *Scolopostethus*. Another known genera of Drymini, e.g. the superficially similar *Salaciola* Bergroth, 1893, which sometimes has similar colour and explanate pronotal carina, are certainly not closely related.

**Etymology.** Patronymic, named after and dedicated to Mallik B. Malipatil, recognizing his excellent contributions to various groups of Australian Heteroptera, particularly Rhyparochromidae. Gender masculine.

**Table 1.** Diagnostic characters of *Malipatilius* gen. n. and *Scolopostethus*.

**Character**	***Malipatilius* gen. n.**	***Scolopostethus***
Eye	hind margin straight	rounded
Antennal segment I surpassing apex of head	short, less than half length of segment	longer, more than half length of segment
Length: width ratio of antennal segment III	~3.5	more than 5
Colour of pronotum	unicolorous dark (sometimes posteriorly slightly paler)	tricoloured
Lateral margin of pronotum	invariably dark	always pale on middle
Pronotal margin at transverse furrow	virtually not widened; strongly concave	distinctly widened, straight or slightly concave
Anterior pronotal lobe of male in side-view	strongly emerging, approximately as high as posterior margin	slightly emerging, nearly evenly sloping
Transversal furrow	deep	shallow
Posterior pronotal margin	straight	concave
Scutellum in side-view	convex	flat
Number of rows of punctures on clavus	3 (inner row sometimes incomplete)	3.5–4
Punctures on corium	even and dense	inner part with smooth parts
Apical margin of corium	straight	slightly S-shaped

## Supplementary Material

XML Treatment for Brentiscerus putoni

XML Treatment for Malipatilius
